# The emotional surge: in-depth qualitative exploration of rumination and emotional turbulence in non-suicidal self-injurers

**DOI:** 10.3389/fpsyg.2024.1449110

**Published:** 2024-12-19

**Authors:** Soulat Khan, Tasnim Rehna, Tayyab Ali Butt

**Affiliations:** ^1^Department of Applied Psychology, National University of Modern Languages (NUML), Islamabad, Pakistan; ^2^Department of Psychology, Foundation University Islamabad (FUI), Islamabad, Pakistan

**Keywords:** non-suicidal self-injury, emerging adults, qualitative study, rumination, distress tolerance, emotional turbulence, negative emotions

## Abstract

Non-suicidal self-injury (NSSI) is an emerging pathological condition among emerging adults, causing significant distress and hindering daily life functioning. The increasing prevalence of NSSI highlights its importance as a crucial area requiring clinical attention. To devise effective interventions for managing NSSI, it is important to identify the factors contributing to its onset and maintenance. Therefore, the current study aims to explore emotional and cognitive factors to provide a holistic understanding of NSSI in emerging adults in the Pakistani context. For this purpose, a qualitative study was conducted using in-depth, semi-structured interviews with *N* = 10 self-injurers (mean age: 22.2 years) recruited through purposive sampling. Participants had engaged in self-injury at least five times in the past year without suicidal intent. The arm was identified as the most common site for self-injury, with cutting being the most frequently used method. Data from the transcribed interviews were analyzed using Braun and Clarke's reflexive thematic analysis. Three main themes—‘emotional turbulence,’ ‘low distress tolerance,’ and ‘rumination’—and nine subthemes emerged, highlighting the emotional and cognitive factors contributing to the initiation and maintenance of NSSI. The findings of this study provide valuable insights into the emotional and cognitive dimensions of NSSI in emerging Pakistani adults. These insights will aid in treatment planning and in selecting appropriate strategies for reducing and ultimately eradicating NSSI.

## Introduction

Emerging adulthood is a life stage characterized by significant biological, sociological, and psychological changes. Transitioning from adolescence to emerging adulthood and embracing new roles can be particularly challenging (Hochberg and Konner, 2020). According to Arnett (2014), emerging adults are typically between 18 and 29 years of age. Willoughby et al. (2021) suggest that this stage is often marked by heightened engagement in risk-taking behaviors. One such risky behavior prevalent during emerging adulthood is non-suicidal self-injury (NSSI; Steinhoff et al., 2020). Emerging adulthood and mid-adolescence are the two stages where NSSI is at its peak with a high prevalence rate (Gandhi et al., 2018). Dierickx et al. (2023) estimated the prevalence rate of NSSI to be approximately 8.4% among emerging adults.

NSSI is a deliberate and intentional destruction of one’s body tissue without any suicidal intent (Nock and Favazza, 2009). The American Psychiatric Association (2022) has given the “Other Conditions That May Be a Focus of Clinical Attention” status in DSM-5-TR. The DSM-5-TR further defines NSSI as intentional damage to any body part without suicidal intent, resulting in pain, bleeding, or bruising through methods such as cutting, hitting, or burning. In a sample of adults in England, cutting was found to be the most frequently used method for inflicting NSSI, followed by burning and swallowing harmful substances or objects (Liu, 2023). NSSI is more prevalent than suicide attempts (Kiekens et al., 2018; Nock et al., 2013), with an onset at an earlier age, and tends to occur more frequently.

Theoretical models of NSSI emphasize the role of cognitive and emotive factors in developing and maintaining NSSI. The cognitive-emotional model of NSSI proposes that those individuals with high emotional reactivity, negative self-concepts, poor emotion regulation, and the belief that NSSI has positive effects in stressful situations are more likely to perceive situations as emotionally volatile and perform NSSI to alleviate their emotional responses. Moreover, cognitions specific to NSSI predispose individuals to self-injure instead of using other emotion regulation strategies to modify their emotional response (Hasking et al., 2017). Another theory, referred to as the Emotional Cascade Model by Selby and Joiner (2009), highlights the role of rumination in NSSI such that individuals with emotional dysregulation undergo an emotional cascade due to which rumination and negative emotions induce behavioral dysregulation, i.e., NSSI (Hatzopoulos et al., 2022). Therefore, rumination fosters negative emotions, resulting in distress ultimately leading to NSSI (Arbuthnott et al., 2015; Selby et al., 2013).

Intense emotional reactions, maladaptive emotion regulation, and inability to identify emotions are the key factors that differentiate individuals involved in NSSI from those who never performed NSSI. Furthermore, more impulse control and acceptance of one’s emotional reactions aided individuals who engaged in self-injury previously in not performing NSSI in the future as compared to current self-injurers (Anderson and Crowther, 2012). Yang et al. (2024) emphasize that less self-control and negative emotions promote NSSI. A study conducted by Voon et al. (2014) explains that stressful life experiences, rumination in the form of anticipatory thoughts and counterfactual thinking, and emotion regulation difficulties have a positive link with NSSI. Thus, repeatedly thinking about life problems is associated with NSSI, strengthening the vicious cycle of engaging in NSSI.

The benefits and barriers model of NSSI, also known as the defective self-model of NSSI, emphasizes the dysfunctional assumptions of defective self-concept and negative views about self, such as feelings of worthlessness and failure, that are held by adults involved in NSSI to validate and regulate their emotions (Hooley and Franklin, 2018). Similarly, Haliczer and Dixon-Gordon (2023) revealed that individuals performing NSSI are more self-conscious and have negative emotional reactions to day-to-day stressful life experiences, leading to psychological distress. Greater self-consciousness and negative emotional reactions predict confusion, NSSI urges, and engagement in NSSI. Boyes et al. (2020) reported that individuals engaged in NSSI have a high intensity of emotions, emotional reactivity, and intensity, and they uphold negative emotions at the trait level.

NSSI has been studied extensively through a qualitative lens in the context of stigma (Meheli and Banerjee, 2022; Rosenrot and Lewis, 2020; Staniland et al., 2021) associated with it and its influence on parents (Fu et al., 2020; Wang et al., 2022). However, there is a lack of knowledge on the psychological antecedents of NSSI. The current study takes into account the experiences of emerging adults engaged in NSSI within the cultural context of Pakistan, providing insight into social and cultural factors leading to NSSI. Moreover, emerging adulthood is a critical age bracket of life transitions, so understanding the issues resulting in self-injury is important. Therefore, the present qualitative study aimed to explore the emotional and cognitive factors leading to NSSI in emerging adults to acknowledge the cultural and psychological underpinnings of NSSI.

## Methods

### Research design

Qualitative research design was used to understand how the individuals engaged in non-suicidal self-injury in Pakistan, specifically its predisposing emotional and cognitive factors, to make sense of their lived experiences. This research design also facilitated the participants to articulate their experiences related to NSSI.

### Sampling strategy and sample

Participants were selected through a purposeful sampling strategy, which allows for the selection of individuals with rich experiences of NSSI (Mapp, 2008). Specifically, intensity sampling, a type of purposeful sampling, was employed to include information-rich cases to study NSSI in detail (Patton, 2014). The sample consisted of *n* = 10 (*n* = 8 females and *n* = 2 males) self-injurers using purposeful sampling who have attempted NSSI at least five times in the past year without the intention of suicide as per DSM-5 TR proposed criteria of NSSI (American Psychiatric Association, 2022). University students meeting the criteria for NSSI were approached and recruited for this study. Participants were selected from Rawalpindi and Islamabad, Pakistan.

The selected age group was emerging adults who were aged 18 to 29 years (Arnett, 2014), as NSSI is prevalent and emerging adults are particularly vulnerable to engaging in it (Kiekens et al., 2023; Mahtani et al., 2019). Emerging adults who injured themselves with suicidal intent were excluded during the sample selection. Emerging adults who were going through the process of bereavement, having a serious medical problem or a terminal illness, or with any physical disability were excluded. Married emerging adults were also not included in the study.

### Procedure

After the research was approved by the Ethics Committee, a purposeful sampling strategy was used to identify participants fulfilling the inclusion and exclusion criteria for this study. The researcher developed an interview guide comprising open-ended questions. The central theme of the questions was the experiences related to NSSI, followed by probing questions for in-depth interviews. Each interview was audio-taped with the consent of the participants, and then interview data was transcribed and consequently analyzed to develop themes. Written consent was obtained from participants who were willing to interview for the present study. The demographic form developed by the researcher was filled by the 10 participants. Face-to-face interviews were conducted with each participant using the semi-structured interview guideline with open-ended and non-leading questions.

*Reflexivity* is a significant part of the research process to make it more transparent and reduce biases. The researcher reflected on her feelings and thoughts throughout the research and after the interviews to avoid any judgments and subjective experiences that might have an influence on the interaction with participants (Olmos-Vega et al., 2023) as the individuals engaged in NSSI might feel criticized if there are any biases or judgments during the interview and research process. *Bracketing* is another process that was incorporated by setting aside prior knowledge and opinions about NSSI. Thus, bracketing aided in eliminating the influence of the researcher’s experiences and knowledge on the research process (Neubauer et al., 2022).

### Ethical considerations

The current study was approved by the Institutional Review Board of the National University of Modern Languages (NUML), Islamabad. As this research involved a vulnerable population of self-injurers, the researcher (psychologist) took in-person interviews and resolved any issues that surfaced on the spot. Informed consent was taken from the participants for audio recording, and recording was stopped when the participants were not comfortable with recording the content, such as details of sexual abuse. Written informed consent was obtained from the participants to publish any potentially identifiable information to be included in this article. The participants were allowed to withdraw from participating in this research at any time. Confidentiality and anonymity were ensured, and participants’ identities were not disclosed to anyone. Pseudonyms, e.g., P1, P2, etc., were given to participants during transcription and analyses to conceal their identity. Before the interview, the participants were informed about the purpose of the study and their right to know the research findings. The researcher only transcribed and analyzed the data, and it was not shared with anyone else.

### Data analysis

Thematic analysis was conducted using a six-phase approach by Braun and Clarke (2019). The inductive approach to thematic analysis emphasizes understanding participants’ experiences and making sense of their world (Braun and Clarke, 2019), which is also the essence of phenomenology. The transcriptions were read multiple times, and the audio recordings were listened to again to remove any errors in the transcriptions. Notes were taken during the process of reading and familiarizing oneself with the data (Phase 1: Familiarization with data). The data was coded based on the latent meaning and the interpretation of the participant’s verbatim. Codes were assigned from the verbatim and phrases by the participants (Phase 2: Generating initial codes). Subsequently, codes reflecting the same meaning were clustered into subthemes and themes (Phase 3: Searching for themes). While reviewing the themes, some codes that seemed to be irrelevant to the theme were discarded, and new codes were added to develop specific themes that reflected the data (Phase 4: Reviewing potential themes). The themes were defined and named concisely relevant to the content and research questions (Phase 5: Defining and naming themes). Lastly, the themes were confirmed and reported (Phase 6: Producing the report).

The *member-checking* method was used to verify results, which improved the credibility of the research (Creswell and Creswell, 2017). The themes that emerged through thematic analyses were discussed with the participants through phone calls, as not all of them were available in person. The participants commented on the description of their cases, and the changes required were incorporated into the final results of this study. Moreover, the reliability and the consistency of the researcher’s approach to analysis, was assured through *inter-coder agreement* for cross-checking codes (Guest et al., 2011). The research supervisor cross-checked the coding done by the researcher in this study, which ensured the reliability of the results.

## Results

Interviews were conducted with 10 self-injuring university students, with a mean age of 22.2 years. The demographic characteristics of the sample are mentioned in Table 1, while the NSSI characteristics of the participants are presented in Table 2. The arm was identified as the most commonly injured body part, and cutting emerged as the most frequently reported method of self-injury. Participants noted that they primarily used readily available tools, such as blades and razors, to inflict injuries.

**Table 1:** Demographic characteristics of participants.

Participant	Gender	Age	Birth order	Family structure
P1	Female	23	1^st^	Joint family
P2	Female	22	3^rd^	Nuclear family
P3	Female	23	1^st^	Joint family
P4	Female	24	1^st^	Joint family
P5	Female	23	2^nd^	Nuclear family
P6	Female	20	2^nd^	Nuclear family
P7	Female	20	2^nd^	Nuclear family
P8	Female	21	2^nd^	Nuclear family
P9	Male	22	3^rd^	Joint family
P10	Male	24	1^st^	Nuclear family

**Table 2:** NSSI characteristics of participants.

Participant	Age	Frequency of NSSI (past 1 year)	Age of onset	Methods of inflicting NSSI	Parts of the body where the injury is induced	Duration of NSSI (in years)
P1	23	10–11 times	17	Cuts with blades Cigarette burns	Arm	6
P2	22	40–50 times	18.5	Scratching by nails Cuts from light bulbs, glass, steel hangers, and sharpener blade	Thighs, belly, arms	3.5
P3	23	8–10 times	19	Safety pin Knife Pinching	Arm	4
P4	24	9–10 times	19	Cuts by nail cutter Cuts by eyebrow razor Scratching Punching wall	Arm, thighs	5
P5	23	10–12 times	18.5	Cuts by blades Cuts by knife Cuts by glass	Arm	4.5
P6	20	12–15 times	16	Headbang Slapping Cuts by a compass needle Scratching	Head	4
P7	20	120 times	13	Skin pulling by nails Cuts with blade Cuts with a compass needle Biting	Around nails, arms, hands	7
P8	21	12–14 times	14	Nails Knife Sharpener blade	Forearm, hands, thighs	5
P9	22	15–18 times	18	Needle, Punching wall	Hands, Arm	4
P10	24	8–10 times	17	Knife, blade, scratching	Arm	7

The results of the thematic analysis are presented in Table 3, highlights three themes that emerged from the analysis of interviews with emerging adults: emotional turbulence, low distress tolerance, and rumination.

**Table 3:** Emotional and cognitive themes and sub-themes of NSSI among emerging adults.

Themes	Subthemes	Initial codes
Emotional turbulence	Emotional reactivity	Anger, impulsiveness, and lack of emotional control, as well as triggering comments from others cause, cause intense emotions.
	Negative emotions	Sadness, anger, frustration
	Alexithymia	cannot understand my emotions, impaired emotional processing, unable to express emotions to others.
	Emotional sensitivity	Reacting to minimal issues, feeling others’ emotions easily, feeling intense emotions.
Low distress tolerance	Low distress tolerance	Cannot handle stressful situations. Everything seems to be overwhelming.
Rumination	Negative thinking	Negative thoughts about self, black-and-white thinking, Anxious thoughts about the future, Thinking about the past and bad memories, Self-criticism.
	Guilt	The guilt of not being able to control things, the guilt of not meeting the expectations of others.
	Thoughts and images of self-injury	Images of patterns of cuts, Thoughts about self-injury, Thoughts that ‘I will not be able to cope without self-injury.’
	Self-pity	Thoughts about being burdened by responsibilities, Questioning myself, “Why me?” if I did nothing wrong, feeling worthless.

### Theme 1: Emotional turbulence

Individuals engaging in NSSI often experience significant challenges in managing their emotions. Their emotional states tend to fluctuate, sometimes becoming disproportionately intense, which can lead to NSSI episodes. Emotional turmoil, characterized by a buildup of predominantly negative emotions, creates a foundation that often precipitates self-injury episodes.

#### Subtheme 1: Emotional reactivity

The emerging adults described experiencing a range of emotions, mostly negative emotions, and high emotional reactivity to stressful situations that lead to NSSI. Anger is the primary emotional reaction resulting from stressful triggers.

“When I get angry, I just go into a different, as I go into a different state. I, I do not see who is in front of me and where I am.” (P6).

“Issue is that if I do not do self-injury then where I should displace it. Even though randomly I have impulsiveness in me like I drive my car so fast I do not have fear that …” (P1).

‘I do not have control over myself and emotions’ was the common notion observed among the individuals engaged in NSSI. In addition to feeling the loss of control over one’s surroundings, a sense of loss of control over one’s thoughts, feelings, and behavior is associated with emotional reactivity and is exhibited in the form of conducting NSSI.

“What I have understood is that there is frustration, I guess. I mean, if sometimes this happens, there is so much happening that I have to sit with myself. Like I mean I cannot control, I mean I want to scream or do something, but when I cannot, then I do it (NSSI).” (P7).

“At first I controlled a lot that no my hand would get hurt, but then I could not control myself.” (P2).

“I think from the start I am angry, so I do it. I am angry with myself and my family all the time. “(P8).

“When I experience extreme anger, so I cannot control and understand things, then I do this (NSSI).” (P9).

#### Subtheme 2: Negative emotions

Participants reported having personalities prone to experiencing negative emotions, such as anger and frequent sadness. Notably, anger emerged as a significant negative emotion reported by all self-injurers in this study, presenting a major barrier to overcoming NSSI.

“I never express to other people that I am in distress or whatever, so when it’s so much inside me, then when its end point for me I can no longer bear it, so I do that (NSSI)…. It’s a negative emotional experience when I am full inside.” (P4).

“They (parents) fight in front of everyone, so negative emotions build up gradually, and I just tried it (NSSI) once so…” (P10).

“Before it, I am angry, I cry, and I am stressed. My emotions are extremely negative. As I told you, even small thing seems negative, and I have flashbacks of …” (P1).

“It (NSSI) does not happen all of a sudden. It takes three to four hours to build up in mind emotionally and be ready.” (P2).

“It starts with crying. During crying, it’s not like… this is the only thing I think about when I am crying.” (P8).

#### Subtheme 3: Alexithymia

Adults engaged in NSSI complain about emotional difficulties, which also account for difficulties in understanding and expressing emotions felt, especially after facing a stressful situation. Therefore, NSSI becomes a mode of expressing and understanding emotions through physical pain, which gives words to their feelings.

“Sometimes you are low, and it’s just weird, but you cannot convey emotions what is happening and why it’s happening…” (P7).

“Before doing this (NSSI), I cannot understand emotions at that time, just I have to do this, do this.” (P6).

“Before NSSI, it’s a mixed state. I cannot understand what to do, and I do not want to cry.” (P4).

“Before doing it, I am numb. You do not understand why you are doing this, what is the reason, or you are feeling nothing. Only there is numbness in your brain.” (P5).

“Till now, I have not understood it (NSSI). When I do not understand myself, that is why I am feeling overwhelmed at this time. For some reason, it provides physical relief to me at that time…. Sometimes, I do not think about why and what I need to do (P8).

#### Subtheme 4: Emotional sensitivity

Being hypersensitive to self and others’ emotions predisposes individuals performing NSSI to recurrently engage in it. Thus, emotional sensitivity exposes the individual to intense emotions, which, coupled with emotion regulation difficulties, contributes to NSSI.

“I am very emotional. Still, I am very much because I am sensitive for myself, but when I see movies, I do not know what happens internally; I have goosebumps.” (P3).

“If any minimal issue occurs; if you ask what happened, it would be a small thing, normal. But then all the things that occurred previously will come to mind…” (P7).

“I do not share my feelings because I am very sensitive, so I do not want to burden myself.” (P4).

### Theme 2: Low distress tolerance

Emerging adults with low distress tolerance struggle with managing emotional distress and stressful events. The low distress tolerance leads to adopting maladaptive coping mechanisms, which include NSSI. Heightened negative emotional experiences and stressful events perceived by the individual, along with low distress tolerance, increase the probability of NSSI.

“Emotions are there at the back of my mind, and if there is a little inconvenience at that time or my father says something, something very small and normal, even then I do it. My emotions are suppressed, so I just made a reason to do it (NSSI).” (P5).

“I have studied, and at the weekend, I have to go out for household work, both things side by side, so you think that this is not my work. My father should do this work, and my younger brother should do it. So, I became angry and did it (NSSI) this way. Recently, this happened again, and I did this (NSSI).” (P1).

“If someone is talking or doing something wrong, that person is saying anything, I cannot tolerate it. Anger issues are so much and tolerance level is very less…” (P2).

### Theme 3: Rumination

The vicious cycle of repetitive thinking and overthinking about negative emotions and adverse events contributes to the continuation of NSSI as a coping mechanism for self-injurers to get control of their negative thoughts about themselves, others, and self-injury as well. Moreover, a stressful event or recollection of memory leads to rumination and aggression associated with a particular event or memory. Rumination includes both repetitive thinking and mental images, which build up negative emotions such as anger, leading to NSSI.

#### Subtheme 1: Negative thinking

Negative thinking was found in emerging adults related to past events and what happened at those moments. Negative thinking about the past as well as the future was present, which increased their anxiety and negative emotions, leading to self-injury.

“It takes two to three days to overthink…. These could be possibilities, or this would happen this way, so this affects self-injury.” (P6).

“Right before self-injury, the events come to my mind in chunks. They are small, but they cluster up and come up collectively.” (P10).

“Sometimes I myself get irritated that why it’s not a big thing, but I am just constantly thinking, thinking and thinking.” (P4).

“I feel out of place. I do not belong here. Nobody likes me, and I have to give something to maintain a friendship.” (P9).

“This happened more due to future anxiety and thinking about it. Now, I do not do self-injury due to external factors, but if something happens, like last time when I got angry, I was not able to understand what to do, so I cut myself. So this was the whole scene… Future anxiety happens because I am in the spotlight. I have to excel.” (P1).

“If you ask me about the issue, it would be minor. But then the things kept on repeating and running in my mind, then everything comes to my mind. If it’s minor, even then this (NSSI) will come up.” (P7).

“Self-hatred is there. It not the way I look, it’s the way I think that why are you looking this way today…. My brother did this, why cannot I do anything.” (P8).

#### Subtheme 2: Guilt

Almost all the participants stated that they have guilt related to their emotions and the events that led to NSSI. Guilt was evident before performing self-injury. Some participants also regard anger to be a personality trait accompanied by self-hate and guilt, resulting in NSSI to process the guilt and validate their feelings.

“In some situations, I did because of guilt…. That why you are crying after something or why I am unable to handle things. This guilt surfaces at that time…” (P5).

“I have the guilt of hurting their (family) expectations… Also, my mother will be embarrassed, and my father will be embarrassed because of my relationship…” (P1).

“I felt guilty (Interviewer: about what?)… I do not know (pause) I do not know, I just feel that maybe things were in my control and I could control them.” (P7).”

“Guilt is there. It’s with myself that why I am like this. I do not even try that ok if someone is saying that you do not talk.” (P4).

“Like you are having a lot of guilt and you are obviously uncomfortable, you are not feeling good, and you get angry so you go and do this (NSSI).” (P10).

#### Subtheme 3: Thoughts and images of self-injury

Participants reported having distressing images and thoughts of NSSI, and they have urges on which they act. To avoid the distressing experience of mentally visualizing self-injury patterns and thoughts of performing NSSI, these individuals engage in NSSI.

“I have imagery of it (NSSI) like I do imagine that I will do it (NSSI) this way. I try to fight with them (images).” (P4).

“I think again and again that I would not be able to cope at that time if I did not do it (NSSI).” (P10).

“…. Suddenly, it comes to my mind that I have to end it, I have to do it (NSSI). It comes automatically to do it, and that night, I had to take the stationary item blade. It was on my side table, so accordingly, it was coming that at that area I have to cut, a proper picture was coming to mind, and it was proper in detail that I have to cut in depth….” (P2).

#### Subtheme 4: Self-pity

Individuals performing NSSI explain the pathway toward NSSI through self-pity. Self-injurers see themselves as misfortunate and as victims, not deserving of what happened to them. These thoughts related to self-pity generate negative emotions, which shape emotional reactivity in the form of anger, resulting in NSSI.

“When I did it for the first time. Thoughts came to my mind that I did not deserve this and why this happened with me, and I had so much pain inside me because I am giving my loyalty to someone…” (P3).

“There are toppers and extra intelligent students in my class. So, I do not know, I feel like I am a loser in front of them …. I am a loser, so I just did. I think that I am nothing, so I do it (NSSI).” (P6).

“I did not have a phone. This is not happening. That thing is not happening. Then I saw friends that they were asking why I did not have it. Then I used to get angry and not eat, which was why I did it (NSSI).” (P1).

## Discussion

The current study incorporated a qualitative approach to explore the antecedents of NSSI in emerging adults in Pakistan. To the best of our knowledge, this is the first indigenous qualitative study focusing on the experiences of emerging adults engaging in NSSI and the psychosocial factors associated with it, providing insights into the cultural aspects influencing NSSI. For this purpose, the thematic analysis was employed, which revealed themes of “Emotional Turbulence,” “Low Distress Tolerance,” and “Rumination,” which were evident in the emerging adults engaging in NSSI.

The theoretical model by Nock (2009) posits that NSSI is a coping behavior for individuals as they have a pessimistic view of themselves and feel that NSSI is the way to regulate their distress. Stressful life events are the main causal factor, with emotional reactivity leading to performing NSSI (Hamza et al., 2021; Nock, 2009) among emerging adults. Moreover, NSSI serves as a potential distractor from negative emotions and thoughts, which in turn aids in improving negative moods, as proposed by the benefits and barriers model of NSSI (Hooley and Franklin, 2018).

Thus, NSSI functions as a means of displacing negative emotions (Muslimah et al., 2024), such as anger, sadness, and crying spells, as reported by participants, which further heightens and leads to NSSI. NSSI has been found to have a strong association with heightened emotional reactivity and emotionality with family dysfunction and social stressors that pose a threat to impulsiveness (Lockwood et al., 2020; Muslimah et al., 2024) and aggression (Buelens et al., 2023; Zhang and Zhang, 2023), leading to NSSI.

Emotional reactivity to negative events often manifests as emotional sensitivity, which has been reported to be higher in women with a history of NSSI (Mettler et al., 2023) as compared to male participants in the present study.

Therefore, individuals who engage in NSSI experience strong, intense emotions (Guerreiro et al., 2013; Hasking et al., 2017; Mettler et al., 2021) and greater interpersonal sensitivity (Masi et al., 2018) than those who do not.

High parental expressed emotions (EE) are a significant factor contributing to emotional turbulence in self-injurers in Pakistan. EE is associated with psychopathology, guilt, feelings of worthlessness, and inadequacy in children, often stemming from the authoritarian parenting style prevalent in Pakistan (Aslam, 2013).

Moreover, there is a significant link between parental expressed emotions and NSSI (Ammerman and Brown, 2018; Baetens et al., 2015; Tschan et al., 2022; Wedig and Nock, 2007), emphasizing the importance of contextual factors such as parenting styles and family dynamics in understanding NSSI in Pakistan.

The dual-harm model of self-harm and aggression focuses on the co-occurrence of self-harm and aggression, which provides a theoretical grounding of aggression to be an emotional response that co-occurs with NSSI (Shafti et al., 2021). Lack of self-control is another significant subtheme that emerged, indicating high emotionality. Other studies also suggest that individuals engaged in NSSI describe feeling limited control over their emotions and themselves. Low self-control hinders an individual’s ability to refrain from engaging in NSSI (Mancinelli et al., 2022). A disempowered state has been identified in NSSI individuals who feel vulnerable and subordinate to others in relationship dynamics (Peel-Wainwright et al., 2021). Thus, NSSI is a tool for regaining control over self and environment, as revealed by the thematic analysis in the current study.

Moreover, the difficulty in articulating feelings is a challenge for self-injurers. Raffagnato’s theory posits that individuals performing NSSI do not have words to state their negative emotions, i.e., alexithymia, and they express these feelings through NSSI (Raffagnato et al., 2020). Similarly, recent quantitative studies accounting for predictors and risk factors of NSSI found alexithymia to play a significant role as a risk factor for NSSI among adolescents (Cao et al., 2024; Dong et al., 2023; Ke et al., 2024; Raffagnato et al., 2023; Zhang et al., 2023). Specifically, the difficulty in identifying their feelings (Ruan et al., 2024) or what is happening to them is a common complaint of self-injurers, which predicts NSSI. However, its relevance is tremendous in the context of Pakistan.

Alexithymia is highly prevalent in young adults in Pakistan. Harsh, often authoritarian parenting and adverse childhood experiences (Karukivi and Saarijärvi, 2014) contribute to difficulties in emotional expression, making it challenging for individuals to articulate their feelings in various situations (Khan and Shabbir, 2019).

Emerging adults engaged in NSSI struggle primarily with emotion regulation and have high positive and negative affectivity, in addition to low distress tolerance, including a negative appraisal of distress and paying more attention to distress-causing situations (Boyd, 2022; Erol and Inozu, 2023; Slabbert, 2021; Slabbert et al., 2021, 2022). Anestis et al. (2013) revealed that facing painful adverse events and experiencing NSSI increases emotion dysregulation, which in turn elevates the rate of suicidal behaviors. This evidence highlights the role of distress tolerance reported by the emerging adults in this study, which further confirms the emotional cascade model, which states that low distress tolerance increases cascades of rumination and negative emotions (Slabbert et al., 2018). Moreover, the Experiential Avoidance Model (EAM) of self-harm, which focuses on avoidance of negative thoughts and emotions in the form of NSSI, further underscores poor distress tolerance as a risk factor and its significance in aiding individuals involved in NSSI in tolerating negative emotions and coping with distress (Chapman et al., 2006). Thus, the theme of low distress tolerance that emerged in this study aligns with the theoretical models.

Studies conducted in Pakistan indicate low distress tolerance among university students, which is associated with maternal rejection (Azhar et al., 2020) and parental overprotection (Saleem et al., 2021). Parenting styles, family rigidity, dysfunctional family dynamics, and family enmeshment are more evident in self-injurers (Khan and Kausar, 2021), highlighting the role of upbringing and family dynamics in the Pakistani context. The self-injurers in the current study were also informed about the family dynamics and the issues associated with them, leading to emotional pain and low distress tolerance.

Another important aspect is rumination in the maintenance of NSSI, backed up by meta-analysis indicating a small effect size and positive relation with NSSI (Coleman et al., 2022; Nagy et al., 2023). The role of rumination in NSSI aligns with the Emotional Cascade Model by Selby and Joiner (2009), which posits that individuals with emotional dysregulation undergo an emotional cascade due to which rumination and negative emotions induce behavioral dysregulation, i.e., NSSI (Hatzopoulos et al., 2022). This also serves as a distraction for these individuals (Selby and Joiner, 2009). Shame and guilt play a role in engaging in NSSI, which fosters self-hatred and low self-esteem (Chakraborty et al., 2023).

Pakistan has a collectivist rather than an individualistic culture, focusing on others’ well-being rather than expressing emotions. Therefore, they appraise their negative emotions and do not acknowledge positive emotions (Azhar et al., 2018), which might reinforce the cycle of ruminating over negative feelings.

Guilt and self-pity have been identified as pivotal factors in amplifying negative emotions and contributing to engagement in NSSI. This phenomenon is particularly relevant in the Pakistani context, given its patriarchal societal structure. There are a lot of expectations from women along with it. They are considered inferior.

Additionally, empirical evidence suggests that guilt, self-pity, and worthlessness are factors commonly manifested as part of depression, which can precipitate NSSI behavior (Hack and Martin, 2018). AghaMohammadi et al. (2024) conducted interviews with self-injurers and found that feelings of worthlessness were prevalent, while guilt was also identified as a significant contributor to NSSI (Serra et al., 2022; Victor et al., 2019).

Mental images and intrusive thoughts of self-injury are distressing for emerging adults, which compel them to perform NSSI. Loss of control is evident during this time, and intrusive mental images are associated with the severity of NSSI (Cloos et al., 2020). The urge reported by self-injurers is linked with mental images of NSSI, mainly illustrated as flash-forward images of self-injury patterns and the relief associated with performing it (Hasking et al., 2018; Ji et al., 2024). Therefore, the thoughts and mental images of NSSI increase the urge to perform it, specifically the anticipated injury pattern. In addition, negative thinking in the form of criticism toward self and hopelessness is distressing for self-injurers. Anticipated injury images were the most common mental imagery reported (McEvoy et al., 2017). A meta-analysis by Lawrence et al. (2023) suggests mental imagery related to NSSI is highly prevalent, along with being instructive, preoccupying, and realistic, as compared to verbal negative thoughts associated with NSSI, which makes the self-injurers more emotionally burdened. Thus, mental images related to self-injury are novel areas explored through interviews with emerging adults, as the NSSI surge is associated with mental images, highlighting their crucial role in the management of NSSI and associated symptoms.

### Limitations and recommendations

This study provides insights into the antecedents of NSSI and the factors associated with its occurrence but does not account for psychiatric conditions such as autism, mood disorders, anxiety disorders, or borderline personality disorder, which may be associated with NSSI. No formal psychological assessment was conducted before selecting the participants. Future studies can conduct formal screening and psychological assessment of individuals engaged in NSSI to rule out comorbid conditions. Secondly, the participants were asked about the frequency and severity of NSSI. A quantitative measure, suitably a rating scale, would give a precise account of the frequency and severity of NSSI, which could be incorporated with the themes identified in this study.

Moreover, the participants of this study are between ages 20 and 24, which is a narrow age bracket due to the homogeneity of the sample. Emerging adults are between 18 and 29 years of age (Arnett, 2014), which limits the generalizability of the results from this study. Therefore, it would be suitable to select participants such that the whole age bracket of emerging adults is covered to increase the generalizability of the results.

## Conclusion

The current study is the first indigenous qualitative investigation into the emotional and cognitive factors contributing to NSSI in emerging adults in Pakistan. The findings highlights the major role of emotional disturbances, rumination, and low distress tolerance in the development of NSSI, highlighting the need to examine parenting practices that may contribute to this behavior in the Pakistani context. Additionally, these maladaptive psychological and emotional processes hinder healthy functioning, prompting individuals to resort to NSSI as a maladaptive coping strategy. Identifying and addressing maladaptive environmental and interpersonal factors is therefore crucial to breaking the self-harm cycle and intervening before NSSI becomes an entrenched coping mechanism and a potential threat.

## Data Availability

The datasets presented in this article are not readily available because the transcripts include the details of the participants, which might be against the anonymity and confidentiality assured to participants. Requests to access the datasets should be directed to Soulat Khan, soulatphd@gmail.com.
